# The Treatment of Strumous Glands in the Neck

**Published:** 1898-10-22

**Authors:** 


### THE TREATMENT OF STRUMOUS GLANDS IN THE NECK.

In discussing the question of the surgical treatment of tuberculous lymphadenitis, especially as occurring in the neck, Dr. Beatson points out that, broadly speaking, the main groups under which this condition presents itself are three in number: (1) A single movable gland; (2) a bunch of movable glands, all of which remain separate and distinct from one another; (3) a large mass of glands matted together and embedded in dense fibrous tissue. When grasped it is felt that the mass, though movable as a whole, has firm and deep attachments, and that the individual glands composing it are blended one with another. Now, in dealing with such cases, in regard to the first two groups it is well to rely on constitutional treatment; but if resolution does not take place, and caseation and softening are imminent, to proceed to incision with complete evacuation of the contents by the sharp spoon, followed by stuffing with Berlin wool impregnated with iodoform, after the manner described by Stiles. In regard to the third group it is a different matter. The first thing to be done in such cases is, of course, to be sure that what we are dealing with is a local tuberculosis of the lymphatic glands, a condition unquestionably of an infective nature, as shown by its passing from gland to gland, and yet not necessarily part of a general tuberculosis. We must also be satisfied that the swelling is not of the nature of a lymphadenoma, nor of those forms of sarcoma known as lymphosarcoma and lymphatic gland sarcoma, and for this purpose a portion may be removed for examination. This point, however, having been cleared up much will depend upon the age of the patient. In young children excision is not to be recommended. Reliance must be placed on caseation and softening of the glands, with subsequent incision and scraping. No doubt some risk of general tuberculosis is run, but very young children do not bear well the lengthy and severe operative measures that such cases undoubtedly entail. It is different, however, with young adults. If they are otherwise in good health, excision is the best treatment for any big mass of enlarged cervical glands that, notwithstanding constitutional treatment, is increasing in size and extent. It must be remembered that the operative measures required are of a somewhat severe character, and may involve, as indeed happened in the case which served as a text for Dr. Beatson's remarks, removal of a portion of the internal jugular vein along with the diseased structures. If we were to make any comment on this teaching, it would be to the effect that the severity of the operation which is sometimes required, when the glands have become agglutinated by secondary inflammation, forms a strong argument in favour of operation at an early stage, even when they are single and distinct, without waiting for caseation and possible infection with pus-producing organisms.

